# Evaluating patients and healthcare professionals' understanding of voting rights for patients in government elections

**DOI:** 10.1192/bjo.2021.796

**Published:** 2021-06-18

**Authors:** Mark Winchester, Madiha Majid, Ashok Kumar

**Affiliations:** Coventry and Warwickshire Partnership Trust

## Abstract

**Aims:**

To understand whether mental health patients vote in government elections

To ascertain the barriers that prevent them from doing so

To explore ways in which mental health services can support patients to vote

To determine whether mental health staff are aware of patients’ right to vote

**Background:**

Members of Parliament (MPs) can influence decisions regarding the National Health Service (NHS) and mental health legislation. The general election on 12th December 2019 highlighted that many patients were not using their democratic right to vote. It also appeared that many staff members were not aware that patients under the Mental Health Act (MHA) were entitled to vote (except for those under ‘forensic’ sections of the MHA). We therefore conducted a survey to ascertain both patient and staff understanding of their democratic rights and to better understand how we could increase the rate of voting amongst psychiatric patients.

**Method:**

Two questionnaires were produced, one for patients and the other for staff members. This was tested by the clinical governance team before approval was granted. Data were collected at the Coventry and Warwickshire Partnership NHS Trust in the form of paper forms or electronically through a survey website. Forty-two patients and twenty-five staff members responded.

**Result:**

No staff members had received formal training with regards to patients’ right to vote. Over half of staff members incorrectly believed that patients under Section 2 or 3 of the MHA and those lacking capacity couldn't vote. More than half of the team members surveyed stated that they had not supported patients in registering or casting a vote. Roughly one third of healthcare professionals felt that it was their responsibility to promote patients’ right to vote, with one third disagreeing and the remaining third unsure.

Over 75% of patients did not vote but less than one quarter of all patients surveyed felt support from mental health services would increase the likelihood of them voting. The main barriers to voting were being mentally unwell, hospital admission or a lack of knowledge on the candidates and election process.

**Conclusion:**

Basic training is required to improve staff knowledge of patients’ voting rights, which should help improve their ability to support patients to vote. Trusts should have a clear protocol in place in the event of future elections, with information on who can vote, how to request a postal vote and the candidates in that area.

